# Commentary: A novel and effective ECG method to differentiate right from left ventricular outflow tract arrhythmias: angle-corrected V2S

**DOI:** 10.3389/fcvm.2023.1167423

**Published:** 2023-07-07

**Authors:** Zhuoqiao He, Ming Liu, Xuerui Tan

**Affiliations:** ^1^ Department of Cardiology, First Affiliated Hospital of Shantou University Medical College, Shantou, China; ^2^ Centre for Precision Health, Edith Cowan University, Perth, WA, Australia; ^3^ Cardiac Function Department, Wuhan Asia Heart Hospital, Wuhan, China; ^4^ Clinical Research Center, First Affiliated Hospital of Shantou University Medical College, Shantou, China

**Keywords:** electrocardiogram algorithms, ventricular arrhythmia, outflow tract, catheterablation, ROC (Receiver operating characteristic curve)

A Commentary on A novel and effective ECG method to differentiate right from left ventricular outflow tract arrhythmias: angle-corrected V2S By He Z, Liu M and Tan X. (2023) Front. Cardiovasc. Med. 10:868634. doi: 10.3389/fcvm.2022.868634

## Introduction

This commentary builds on the recently published original paper of Qiu et al. (1). The most common origin of premature ventricular complexes and ventricular tachycardias of patients in the absence of structural heart disease is the right and left ventricular outflow tracts (RVOT/LVOT). Catheter ablation (CA) has been an effective method for outflow tract ventricular arrhythmias (OTVAs) (2). It is necessary to predict the origin of OTVAs before CA for choosing the procedural strategy, reducing complications, and saving the operation time (3, 4). Qiu et al. presented a novel electrocardiogram (ECG) algorithm, “cardiac rotation-corrected” angle-corrected V2S (hereafter, V2S angle), to differentiate the LVOT origin from the RVOT origin with higher predictive accuracy (1).

**Figure 1:**
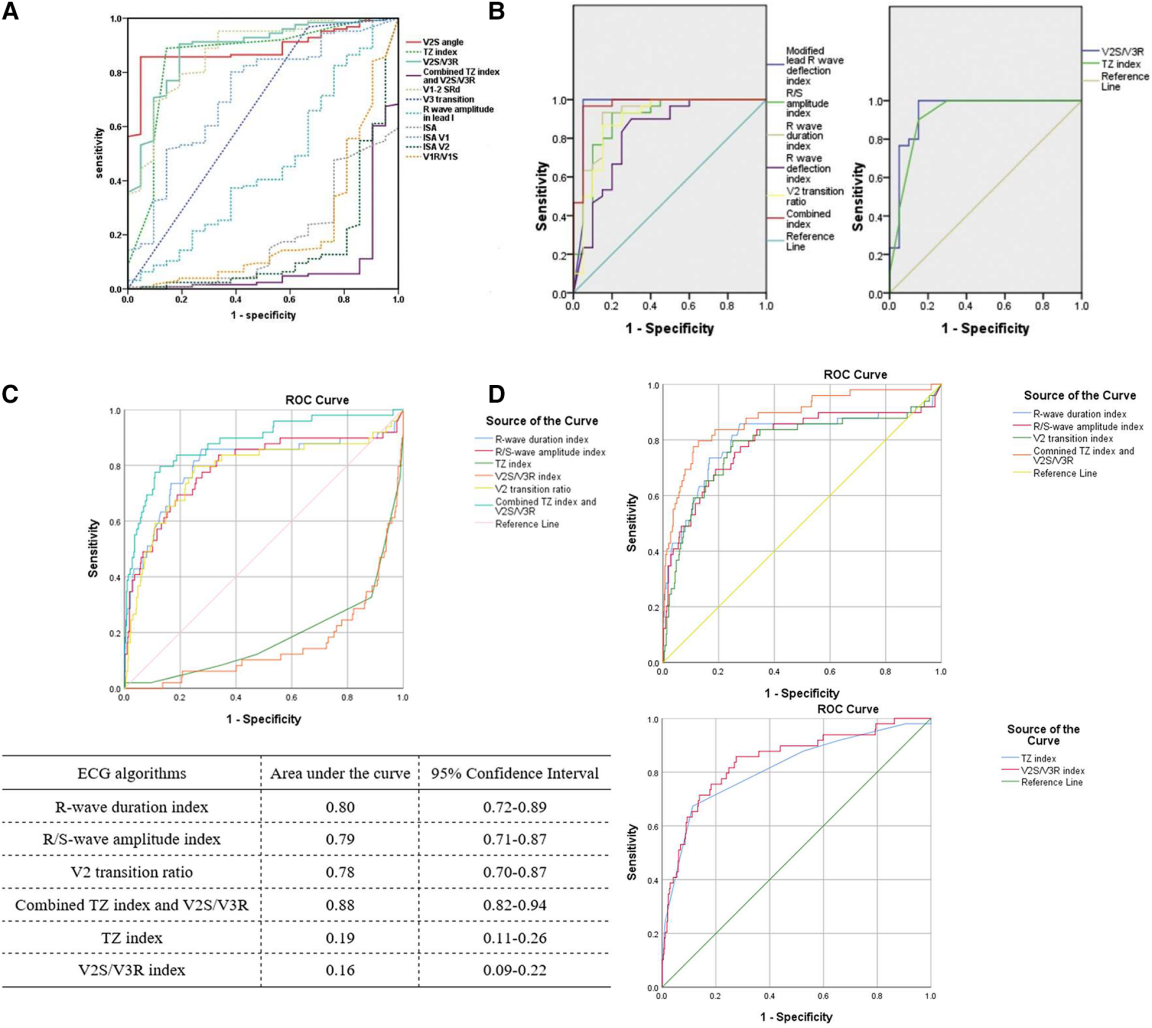
Predictive accuracy of different ECG algorithms from Qiu et al. (**A**) (1), Anderson et al. (**B**) (8), and our previous study (**C**,**D**) (5). (**A**) The ROC curves of the TZ index and V2S/V3R index were above the reference line (diagonal line), while the ROC curve of the combined TZ index and V2S/V3R was below the reference line in the same ROC curve analysis. (**B**) The ROC curves of the TZ index, V2S/V3R index, and combined TZ index and V2S/V3R were all above the reference line in two separate ROC curve analyses. (**C**) Using our published data to compare all the ECG algorithms in the same ROC curve analysis without changing the “Test Direction” for the TZ index and V2S/V3R index, the ROC curves of the TZ index and V2S/V3R index were below the reference line. (**D**) After changing the “Test Direction” (“Smaller test result indicates more positive test”) for the TZ index and V2S/V3R index, the ROC curves of all the ECG algorithms could be above the reference line in two separate ROC curve analyses. (RightsLink License Number 5579081364648).

In the original manuscript, Qiu et al. compared the predictive effects of the V2S angle and several existing ECG algorithms, including the V1R/V1S index, initial R-wave surface area (ISA) index, combined transitional zone (TZ) index and V2S/V3R, R-wave amplitude in lead I, V2S/V3R index, TZ index, and V1-2 S-R difference (V1-2 SRd) using receiver operating characteristic (ROC) curve analysis with the area under the curve (AUC). The AUC of the V1R/V1S index, ISA index, combined TZ index and V2S/V3R, and R-wave amplitude in lead I was less than 0.5, while the AUC of the V2S angle, V2S/V3R index, TZ index, and V1-2 SRd was more than 0.5, according to Qiu et al. (Figure 1A). The ROC curve analysis was conducted using SPSS (SPSS Inc., Chicago, IL, USA). All ECG algorithms were compared in the same ROC curve analysis in the study of Qiu et al. (1).

## Discussion

However, we found it inappropriate to compare all ECG algorithms in the same ROC curve analysis, which placed several ROC curves below the reference line (diagonal line) with an AUC of less than 0.5 (Figure 1A) (1). Fawcett (2006, p. 868) defined an ROC curve as “a two-dimensional depiction of classifier performance” (6). A common approach to compare classifiers is the AUC. The value of the AUC is always between 0 and 1.0 because it is a part of the area of the unit square. However, the AUC of any realistic classifier should not be less than 0.5 because the diagonal line between (0, 0) and (1, 1) produced by random guessing has an area of 0.5 (6). According to Qiu et al., the V2S angle, V2S/V3R index, TZ index, and V1-2 SRd predicted an LVOT origin when their cut-off values were less than 58.28, 1.5, 0, and 1.625, respectively, with AUCs larger than 0.5 (1). Taking the V2S angle as an example, it was indicated that OTVAs were more likely originated from the LVOT when the V2S angle was less than 58.28, namely, smaller value of the V2S angle indicated a more positive test (LVOT origin). The combined TZ index and V2S/V3R, R-wave duration index, R/S-wave amplitude index, ISA index, and R-wave amplitude in lead I predicted an LVOT origin when their cut-off values were larger than −0.76, 0.5, 0.3, 15, and 0.1, respectively, with AUCs less than 0.5 (1). Taking the combined TZ index and V2S/V3R as an example, it was indicated that OTVAs were more likely originated from the LVOT when the value of the combined TZ index and V2S/V3R was larger than −0.76, that is, a larger value of the combined TZ index and V2S/V3R indicated a more positive test (LVOT origin) (5). “Larger value” and “smaller value” indicate that the criteria to determine the positive test are different, which is determined in the “Test Direction” area of the “ROC Curve: Options” dialog in SPSS. Therefore, we should choose a different “Test Direction” of the “ROC Curve: Options” dialog when using SPSS to perform ROC curve analysis. According to the default setting for the “Test Direction” (“Larger test result indicates more positive test”), the likelihood of the State event increases with increasing Test variables (7). Therefore, we should choose “Larger test result indicates more positive test” when choosing the “Test Direction” for ROC curve analysis of the combined TZ index and V2S/V3R, R-wave duration index, R/S-wave amplitude index, ISA index, and R-wave amplitude in lead I, while choosing “Smaller test result indicates more positive test” for ROC curve analysis of the V2S angle, V2S/V3R index, TZ index, and V1-2 SRd. As a result, all ROC curves of the different ECG algorithms can be above the reference line and in the upper left corner with all AUCs higher than 0.5, which is similar to the results of Anderson et al. (Figure 1B). The AUCs of the combined TZ index and V2S/V3R, V1R/V1S index, ISA index, and R-wave amplitude in lead I predicting an LVOT origin may change to 0.912, 0.784, 0.814, and 0.543, respectively, instead of 0.08, 0.216, 0.186, and 0.457 in Qiu's study after changing the “Test Direction”. The ROC curve dipping into the right-hand lower half of the graph indicated that the “Test Direction” area of the “ROC Curve: Options” dialog had been specified in the wrong direction (7). Anderson et al. compared the predictive accuracy of different ECG algorithms in two separate ROC curve analyses (8). They compared the R/S-wave amplitude index and R-wave duration index, with the combined TZ index and V2S/V3R in the same ROC curve analysis due to its consistency in the “Test Direction” (“Larger test result indicates more positive test”). The R/S-wave amplitude index, R-wave duration index, and the combined TZ index and V2S/V3R predicted an LVOT origin when their cut-off values were larger than 0.3, 0.5, and −0.76, respectively. In addition, Anderson et al. compared the V2S/V3R index with the TZ index in another ROC curve analysis because both had the same “Test Direction” (“Smaller test result indicates more positive test”). The V2S/V3R index and TZ index predicted an LVOT origin when their cut-off values were less than 1.5 and 0, respectively. Therefore, the ROC curves of all these ECG algorithms were above the reference line, with all AUCs greater than 0.5 (Figure 1B) (8).

We had previously compared the predictive accuracy of several ECG algorithms, including the R-wave duration index, R/S-wave amplitude index, V2S/V3R index, TZ index, and combined TZ index and V2S/V3R (5). When we compared all the ECG algorithms in the same ROC curve analysis without changing the “Test Direction” for the TZ index and V2S/V3R index, the AUCs of the TZ index and V2S/V3R index were less than 0.5 (TZ index: 0.19, V2S/V3R index: 0.16) using our previously published data (Figure 1C). After changing the “Test Direction” (“Smaller test result indicates more positive test”) for the TZ index and V2S/V3R index, the AUCs could be larger than 0.5 (TZ index: 0.81, V2S/V3R index: 0.84) (Figure 1D) (5).

## Conclusion

In conclusion, when calculating the AUCs of different ECG algorithms in the ROC curve analysis using SPSS, the “Test Direction” area of the “ROC Curve: Options” dialog should be selected according to the content of the ECG algorithms (“Larger or smaller test result indicates more positive test”). The AUC of any realistic classifier should not be less than 0.5. A dip in the ROC curve in the lower right corner of the graph could indicate that the “Test Direction” area has been specified in the wrong direction.
